# Connections between Classical and Parametric Network Entropies

**DOI:** 10.1371/journal.pone.0015733

**Published:** 2011-01-05

**Authors:** Matthias Dehmer, Abbe Mowshowitz, Frank Emmert-Streib

**Affiliations:** 1 Institute for Bioinformatics and Translational Research, UMIT, Hall in Tirol, Austria; 2 Department of Computer Science, The City College of New York (CUNY), New York, New York, United States of America; 3 Computational Biology and Machine Learning, Center for Cancer Research and Cell Biology, School of Medicine, Dentistry and Biomedical Sciences, Queen's University Belfast, Belfast, United Kingdom; University of East Piedmont, Italy

## Abstract

This paper explores relationships between classical and parametric measures of graph (or network) complexity. Classical measures are based on vertex decompositions induced by equivalence relations. Parametric measures, on the other hand, are constructed by using information functions to assign probabilities to the vertices. The inequalities established in this paper relating classical and parametric measures lay a foundation for systematic classification of entropy-based measures of graph complexity.

## Introduction

Information theory has proven to be a useful tool in the analysis and measurement of network complexity [1]. In particular, many researchers have investigated the application of entropy measures to graphs, see [1]–[4]. A variety of entropy-based measures have been used to characterize networks associated with biological or chemical systems [5], [6]; a recent application in computational biology uses an entropy measure to analyze metabolic networks [7], [8]. In addition to the use of measures on graphs to analyze biological or chemical systems, information theory has been employed in network physics, see [1], [9], [10]. Arnand et al. [1] provide a comprehensive review of Shannon entropy measures applied to network ensembles. The measures discussed in this review are based on probabilistic aspects of networks such as the expected number of links, the expected community structure, or the expected degree sequences that have been used to define probabilities. In addition, Arnand et al. [1] compared Shannon entropies on network ensembles with Gibbs and von Neumann entropies by plotting numerical values of the respective measures. By contrast, we will establish connections between different definitions of entropy by means of inequalities. Sanchirico et al. [10] explored another problem in network theory, namely, characterizing complex networks based on degree distributions. In particular, entropy functions have been used to investigate scale-free networks, see [10]. Finally, Krawitz et al. [9] have applied the so-called Basin entropy to boolean networks, which have been shown to be of great value in analyzing biological [7] and related systems [9]. Krawitz et al. [9] applied the Basin entropy measure to specific components of boolean networks [9]. In these applications, entropy provides a measure of network connectivity. It is noteworthy that Krawitz et al. [9] were able to estimate the Basin entropy from time-series data, since the model thus becomes applicable to erroneous networks (i.e., graphs affected by measurement errors) which are of great importance in biology.

The information measures we want to consider in this paper represent the structural information content of a network [5], [11]–[13]. In particular, they have been applied to special classes of graphs and have figured prominently in research on topological aspects of biological and chemical systems, e.g., see, [5], [11]–[18]. Common to all such research is the use of Shannon's [19] classical measure to derive entropies of the underlying graph topology interpreted as the structural information content of a graph. [5], [11]–[13]. Measures of this kind are functions of probability values that derive, in the classical case [5], [12], from a graph invariant and an equivalence relation [11], [12], [20]. Thus far, a number of specialized measures have been developed that are used primarily to characterize the structural complexity of chemical graphs [11], [21], [22]. That is to say, these measures can be viewed as indexes of complexity based on certain structural features of a graph. In the classical cases, special graph invariants (e.g., number of vertices, edges, degrees, distances etc.) and equivalence relations have given rise to special measures of information contents [11], [12], [15].

Another class of graph entropies, not based on a graph invariant associated with an equivalence relation, has also been explored. These alternative measures are based on information functions [23] that assign a probability value to each vertex of a graph [23]. An interesting feature of these measures is that they are parametric, see, e.g., [2], [15], thus allowing the formulation of optimization problems involving the parameters for given data sets. This approach to measurement is applicable to research problems in graph complexity, data analysis, and machine learning. Furthermore, the measures are computable in polynomial time because they depend on determining metrical properties of graphs [24]. In view of the large number of existing quantitative measures of network structure [22], [25], methods are needed for comparing the different indexes and investigating their interrelations. Such research on interrelations can be expected to yield new insights into complex systems that can be represented by graphs [22], [26].

One promising direction is to infer inequalities between such indices describing network information contents. Relatively little work on this problem has appeared in the literature, see, e.g., [27], [28]. Thus far we have studied in [2] so-called implicit information inequalities involving two parametric entropies using different information functions. General as well as special graphs have been considered [2]. The present paper deals mainly with inequalities between classical and parametric entropies. On the one hand, this gives rise to general information inequalities between measures; on the other hand, bounds for special classes of graphs can be obtained.

The paper is organized as follows: In Section ‘Methods and Results’, we describe the classes of information measures to be examined, and detail relevant properties. Also, we prove inequalities between classical and parametric entropies. The last section provides a summary and conclusion.

## Methods and Results

### Classical Measures and Parametric Graph Entropies

In this section, we sketch briefly known graph entropy measures for determining the information content of networks. As a preliminary remark, 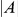 denotes the cardinality of a given set 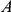. Now, let 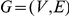 be a graph and let 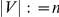. The existing graph entropy measures can be divided into two main classes: (i) Classical measures [14] and (ii) parametric measures [23]. Classical measures 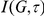 are defined relative to a partition of a set 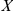 of graph elements induced by an equivalence relation 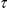 on 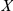. More precisely, let 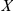 be a set of graph elements (typically vertices), and let 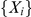 for 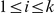, be a partition of 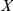 induced by 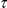. Suppose further that 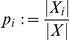. Then
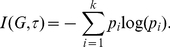
(1)


Parametric measures are defined on graphs relative to information functions. Such functions are not identically zero and map graph elements (typically vertices) to the non-negative reals. For simplicity of description, we consider information functions defined on 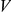. Let 
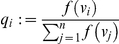
 for 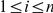. Clearly, the 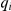 form a probability distribution over the vertices. Then
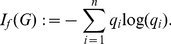
(2)


In general, a detailed overview of graph entropy measures can be found in [2], [11], [14]. Note that various other graph entropies have also been developed, see, e.g., [3], [4], [29]–[32] but these won't be discussed here.

The structural feature of a network captured by a classical information measure depends on the graph invariant and the equivalence criterion 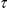. This is clear from Equation (1). The relationship between the quantitative measure and graph structure for classical measures is examined further by Nikolić [33].

For more general measures (Equation (2)), the structural feature depends on the information function used to define the measure. Examples are given by
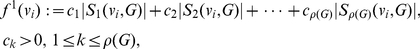
(3)

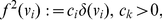
(4)

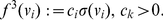
(5)


The 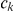 are positive coefficients used to weight structural differences in a graph [23] and 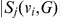 are the 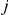-sphere cardinalities. 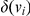 denotes the degree and 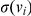 the eccentricity of the vertex 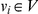. 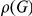 stands for the diameter of 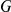. Such functions are used to obtain the vertex probabilities as explained in [23]

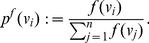
(6)


**Figure 1:**
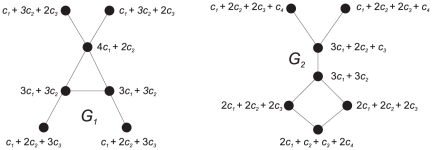
We obtain 2.78 =  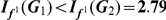.

The family of graph entropies resulting from different probability distributions is represented by Equation (2). In the following, we provide examples of such an information function (choosing 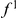 as a special case) as well as of the resulting entropy measure. Furthermore, we compare this measure with a classical one using an identity graph as an example. Note that the information function 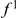 has already been used to characterize chemical structures [15]. But first consider the graphs in Figure 1 to explain the graph entropy measure 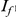 in more detail. For calculating this structural information content explicitly, we set
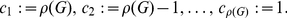
(7)


**Figure 2:**
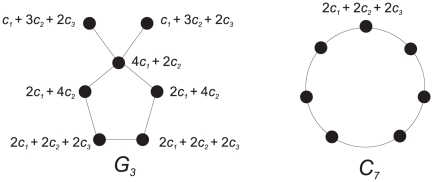
We obtain 2.79 =  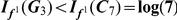.

The structural feature captured by this measure is linked to the following observation: The more the vertices differ with respect to their spherical neighborhoods, the smaller is the value and conversely. Hence, 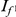 detects a kind of inner symmetry of an underlying graph. By using 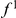 in Equation (2), regular graphs have a constant information content equal to the maximum entropy (for every information function). For example, the graph 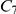 gives rise to (see Figure 2)
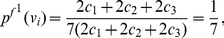
(8)and finally 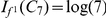. To compare the parametric with one of the classical measures, we consider a special case of Equation (1) in which the probabilities are determined by the respective sizes of the (vertex) orbits of the automorphism group, see [12]. The resulting graph entropy measure is denoted by 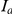. Because 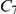 is vertex-transitive, there is only one orbit containing all vertices and therefore we obtain
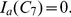
(9)


**Figure 3:**
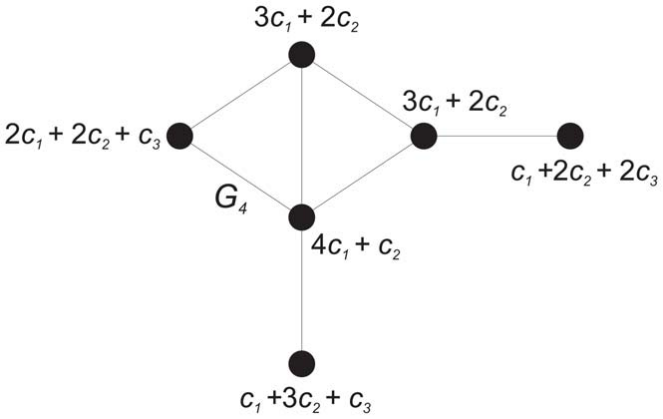
A graph with identity group.

Now, we evaluate the two different graph entropy measures 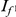 and 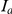 for the identity graph depicted in Figure 3. This graph 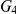 has a trivial automorphism group (i.e., the identity graph) and, hence, all orbits are singleton sets. This implies
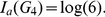
(10)


**Figure 4:**
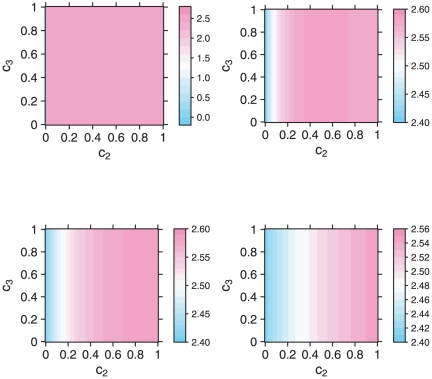
Entropy vs. Parameter Values.

But when calculating 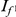, we get
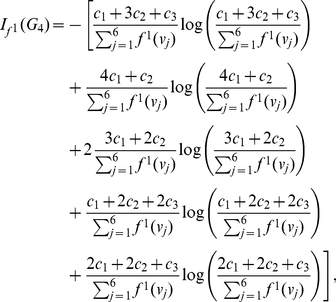
(11)where 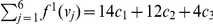. Finally, we find that 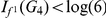. In contrast, note that 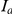 represents a symmetry-based complexity measure [12]. Other structural features could be chosen to provide other or more comprehensive measures of complexity. For example, Bertz [34] modified the total information content discussed by Bonchev [11] to obtain a different measure. Other approaches to tackle this challenging problem have been outlined by Nikolić [33]. To better understand the measure 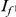 and to get an intuitive sense of its complexity, we perform a parameter study. More precisely, we show the entropy represented by Equation (11) for different parameters. We plotted the entropy for constant values of 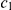 (0, 0.5 - first row, and 1, 3 - second row) independent of the other variables 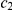 and 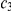, see Figure 4. Clearly, the positions of maximum entropy are shifted for different values of 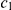; and for higher values of 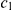 the location of the maximum approaches that of 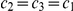.

### Inequalities for Parametric Graph Entropies and Classical Measures

Most of the graph entropy measures developed thus far have been applied in mathematical chemistry and biology [2], [11], [14]. These measures have been used to quantify the complexity of chemical and biological systems that can be represented as graphs. Given the profusion of such measures, it is useful, for instance, to prove bounds for special graph classes or to study interrelations among them. Such results might be useful to investigate network-based systems as well as to design new network measures more adequately. In terms of information-theoretic measures for graphs, relatively little attention has been paid to this effort. An early attempt in this direction was undertaken by Bonchev [27] when investigating inequalities between entropy-based network measures by considering special graph classes. In particular, Bonchev [27] used such inequalities to investigate the concept of branching [35] in molecules. A topic within this general framework which seems to be completely unexplored is an analysis (using inequalities) of formal relations between complexity measures. On the one hand, this could be done by starting from special graph classes which are interesting for practical applications. But, on the other hand, one can also infer more general interrelations between non-information-theoretic and information-theoretic measures (e.g., see Theorem (1)).

In [28], we have investigated so-called implicit information inequalities for graphs. Such information inequalities describe general interrelations between parametric measures based on arbitrary information functions. In this section, we demonstrate inequalities between classical graph entropies and the entropy families given by Equation (2). As mentioned earlier, numerous network information measures [11], [14], [22] have been developed, but their mathematical properties have yet to be studied in detail. Therefore, the results of this section can be considered as a first attempt to detail these properties. Some of the interrelations represent bounds which hold for special graph classes (with no assumptions about the parameters involved) when using a special information function.

We start with a more general statement expressing an interrelation between the parametric entropy and a classical entropy measure that is based on certain equivalence classes associated with an arbitrary equivalence relation. In particular, this interrelation can be stated as an upper bound of the parametric entropy depending on the classical entropy measure.

#### Theorem 1

*Let* 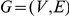 *be an arbitrary graph, and let* 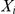 *,* 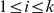 *be the equivalence classes associated with an arbitrary equivalence relation on* 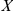 *. Suppose further that* 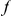 *is an information function with* 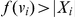 *for* 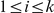 *,* 
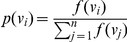
 *and* 
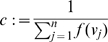
 *. Then,*

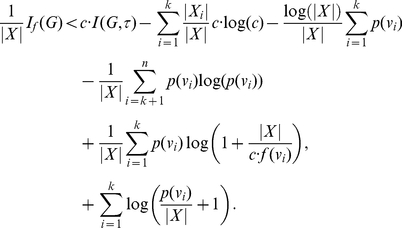
(12)


#### Proof

Note that we use the simplified notation 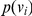 instead of 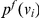 because it is clear (by definition) that a vertex probability value depends on the information function 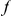. Now, suppose 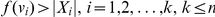. Then,
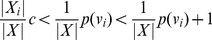
(13)and
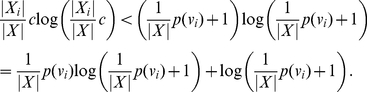
(14)Assuming
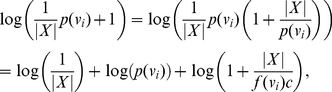
(15)and making use of Inequality (14) we derive
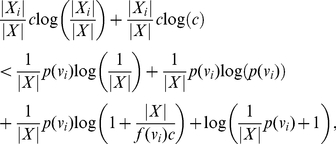
(16)or
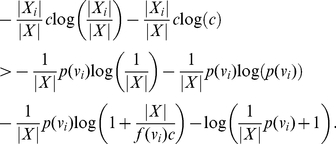
(17)


Adding up these inequalities (i.e., by adding across the vertices), we obtain
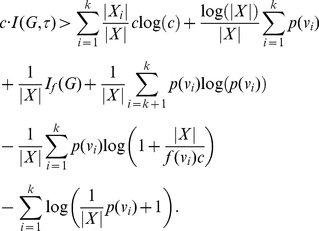
(18)


But this is Inequality (12).

In the following, we apply the assumption 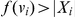 for 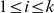 to some special graph classes and using the proof technique of the previous theorem. The set 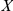 is taken to be 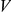, and thus the equivalence relation induces a partition of 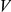 into equivalence classes of vertices. These assumptions allow for obtaining upper bounds on 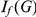 which can be stated as corollaries of Theorem (1).

#### Corollary 2

*Let* 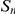 *be a star graph having* 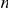 *vertices and suppose* 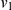 *is the vertex with degree* 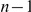 *. The remaining* 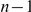 *non-hub vertices are labeled arbitrarily.* 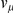 *stands for a non-hub vertex. Let* 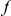 *be an information function satisfying the conditions of Theorem (1). Let* 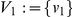 *and* 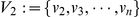 *denote the orbits of the automorphism group of* 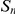 *forming a partition of* 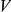 *. Then*

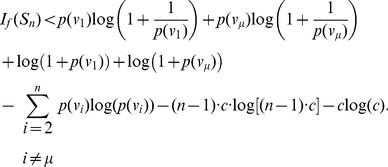
(19)


#### Proof

By hypothesis 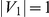 and 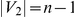 so that
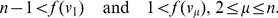
(20)


The information functions given by Equation (3), (4), (5) can be seen to satisfy the above conditions by choosing appropriate 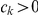. Again, with 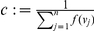, the Inequalities (20) yield
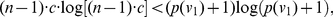
(21)

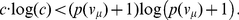
(22)


Now, applying the proof technique of Theorem (1) and performing some elementary transformations, we obtain Inequality (19).

#### Corollary 3


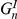
 *be an identity graph having* 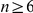 *vertices.* 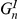 *has only the identity automorphism and therefore each orbit is a singleton set, i,e.,* 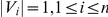 *. Let* 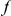 *be an information function satisfying the conditions of Theorem (1). Then,*

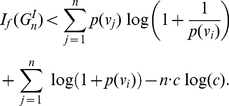
(23)


#### Proof

By hypothesis
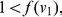
(24)

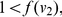
(25)

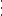


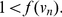
(26)


Clearly,
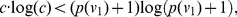
(27)

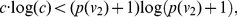
(28)

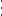


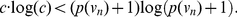
(29)


Once again applying the proof technique of Theorem (1) and performing some elementary transformations, we obtain Inequality (23).

Corollary (3) leads immediately to

#### Corollary 4

*Let* 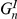 *be an identity graph having* 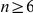 *satisfying the conditions of Corollary (3). Then,*

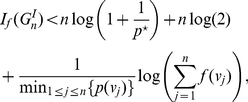
(30)
*where* 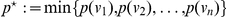.

#### Corollary 5

*Let* 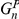 *be a path graph having* 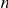 *vertices and let* 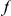 *be an information function satisfying the conditions of Theorem (1). If* 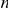 *is even,* 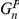 *possesses* 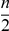 *equivalence classes* 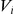 *and each* 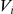 *contains 2 vertices. Then,*

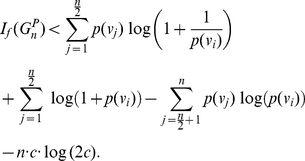
(31)


*If* 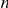 *is odd, then there exist* 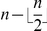 *equivalence classes* 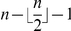 *that have 2 elements and only one class containing a single element. This implies,*

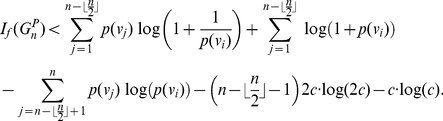
(32)


#### Proof

By hypothesis
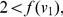
(33)

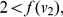
(34)

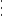


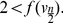
(35)


Then, it is easy to see that
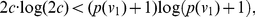
(36)

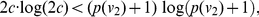
(37)

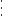


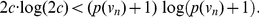
(38)


When 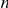 is odd, we have
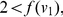
(39)

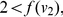
(40)

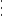


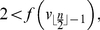
(41)

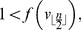
(42)and
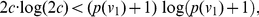
(43)

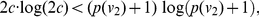
(44)

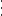


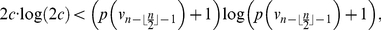
(45)

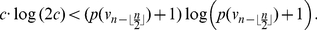
(46)


Multiplying these inequality systems by -1 and performing the addition step (of the proof technique of Theorem (1) gives Inequality (31) and Inequality (32).

Assuming different initial conditions, we can derive additional inequalities between classical and parametric measures. We state the following theorems without proofs because the underlying technique is similar to the proofs of the previous assertions.

#### Theorem 6

*Let* 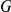 *be an arbitrary graph and* 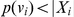 *. Then,*

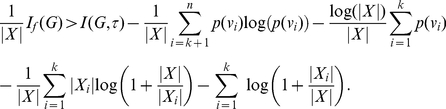
(47)


#### Theorem 7

*Let* 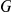 *be an arbitrary graph with* 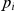 *being the probabilities satisfying* *Equation (1)* *such that* 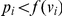 *. Then,*

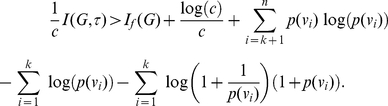
(48)


For identity graphs, we can obtain a general upper bound for the parametric entropy.

#### Corollary 8

*Let* 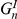 *be an identity graph having* 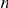 *vertices. Then,*

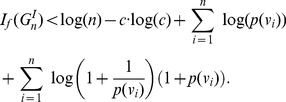
(49)


## Discussion

Quantitative measures of network structure have been defined and applied in many different settings, see, e.g., [2], [22], [25]. For example, chemists represent molecules as graphs as an aid in distinguishing molecules and cataloguing their properties [36], [37]; biologists model natural phenomena as complex networks in the study of brains and genetic information systems [38], [39]; epidemiologists and computational biologists investigate the spread of diseases in populations modeled as networks of individuals [40], [41]; computer scientists design and build networks of artificial systems that support message exchange and distributed computation [42], [43]. In each of these different settings, questions about the structure of networks arise, leading to the definition of mathematical functions designed to measure structural complexity. As a result of all these relatively independent scholarly efforts, many different measures [2], [22], [25], [33] have been defined whose interrelations remain to be determined. This paper is intended as a contribution to the classification of these diverse measures of network structure. In particular, we have singled out two different classes of measures, namely classical and parametric entropy measures defined on graphs, and have examined their interrelations.

The approach taken in this paper is to establish inequalities between measures. As already mentioned, an early attempt in this direction has been undertaken by Bonchev [27] who proved inequalities in the course of investigating branching structures in molecules. Our aim here is somewhat broader, namely to establish general, formal relations between complexity measures defined on arbitrary graphs. Since complexity measures typically assign real numbers to networks, inequalities provide the foundation for constructing partial orders on sets of measures. Knowledge of such order relations enables us to use inequalities to infer bounds on specific measures of the structural complexity of graphs and networks. Knowing that measure 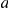 is bounded above by measure 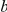 whose maximum value has been established tells us that measure 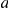 has a maximum value less than that of measure 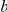. Relatively little work on the problem of ordering entropy measures has appeared in the literature, see, e.g., [44], [45].

The main focus of the paper has been on establishing inequalities between entropy-based measures, i.e., measures that make use of Shannon's classical entropy function. In particular, we examined inequalities involving classical and parametric information measures. Such measures have been used extensively to quantify the information content of systems represented by graphs [2], [12], [21], [22]. For this reason, we believe that such inequalities are critical for a proper understanding of entropy-based measures.

The inequalities presented in this paper show interrelations between entropy-based measures applied to special classes of graphs. Establishing such inequalities for arbitrary graphs is a daunting task. The work reported here is thus a first step in that the methods employed can in principle be generalized to establish inequalities between information measures on arbitrary graphs. More research is clearly needed to extend the results to graphs in general, and ultimately to build a foundation for a unified interpretation of network complexity as measured by entropy-based functions.
